# Evaluation of Hyperpolarized [1-^13^C]-Pyruvate by Magnetic Resonance to Detect Ionizing Radiation Effects in Real Time

**DOI:** 10.1371/journal.pone.0087031

**Published:** 2014-01-27

**Authors:** Vlad C. Sandulache, Yunyun Chen, Jaehyuk Lee, Ashley Rubinstein, Marc S. Ramirez, Heath D. Skinner, Christopher M. Walker, Michelle D. Williams, Ramesh Tailor, Laurence E. Court, James A. Bankson, Stephen Y. Lai

**Affiliations:** 1 Bobby R. Alford Department of Otolaryngology, Head and Neck Surgery, Baylor College of Medicine, Houston, Texas, United States of America; 2 Department of Head and Neck Surgery, The University of Texas M. D. Anderson Cancer Center, Houston, Texas, United States of America; 3 Department of Radiation Oncology, The University of Texas M. D. Anderson Cancer Center, Houston, Texas, United States of America; 4 Department of Imaging Physics, The University of Texas M. D. Anderson Cancer Center, Houston, Texas, United States of America; 5 Department of Pathology, The University of Texas M.D. Anderson Cancer Center, Houston, Texas, United States of America; 6 Department of Radiation Physics, The University of Texas MD Anderson Cancer Center, Houston, Texas, United States of America; 7 Department of Molecular and Cellular Oncology, The University of Texas M.D. Anderson Cancer Center, Houston, Texas, United States of America; Instituto de Investigación Sanitaria INCLIVA, Spain

## Abstract

Ionizing radiation (IR) cytotoxicity is primarily mediated through reactive oxygen species (ROS). Since tumor cells neutralize ROS by utilizing reducing equivalents, we hypothesized that measurements of reducing potential using real-time hyperpolarized (HP) magnetic resonance spectroscopy (MRS) and spectroscopic imaging (MRSI) can serve as a surrogate marker of IR induced ROS. This hypothesis was tested in a pre-clinical model of anaplastic thyroid carcinoma (ATC), an aggressive head and neck malignancy. Human ATC cell lines were utilized to test IR effects on ROS and reducing potential *in vitro* and [1-^13^C] pyruvate HP-MRS/MRSI imaging of ATC orthotopic xenografts was used to study *in vivo* effects of IR. IR increased ATC intra-cellular ROS levels resulting in a corresponding decrease in reducing equivalent levels. Exogenous manipulation of cellular ROS and reducing equivalent levels altered ATC radiosensitivity in a predictable manner. Irradiation of ATC xenografts resulted in an acute drop in reducing potential measured using HP-MRS, reflecting the shunting of reducing equivalents towards ROS neutralization. Residual tumor tissue post irradiation demonstrated heterogeneous viability. We have adapted HP-MRS/MRSI to non-invasively measure IR mediated changes in tumor reducing potential in real time. Continued development of this technology could facilitate the development of an adaptive clinical algorithm based on real-time adjustments in IR dose and dose mapping.

## Introduction

Ionizing radiation (IR) is commonly employed in the treatment of most solid head and neck tumors as definitive therapy or in the adjuvant setting [1], [2], [3], [4], [5]. Unfortunately, radiation toxicity, manifested as mucositis, xerostomia, dysphagia and dysphonia, limits the maximum tolerated dose [6], [7]. Successful IR treatment is therefore driven by its intrinsic therapeutic index, or the ratio of tumoricidal effects to normal tissue toxicity. At the present time, neither parameter can be measured *in vivo*, in real-time, in order to appropriately adjust treatment based on actual tumor response. Therefore, total dose, dose fractionation and dose painting decisions are largely based on empirical data derived from previous prospective and retrospective trials [1], [8], [9], [10], [11]. Chen et al. recently reported that metabolic imaging using hyperpolarized tracers (pyruvate) can detect changes in tumor viability within days of irradiation [12]. Their exciting data suggest that metabolic imaging may be a useful clinical tool in adjusting irradiation protocols in order to maximize clinical effectiveness. In this study, we present evidence, which indicates that the useful window for metabolic imaging can be extended to within hours of irradiation, further enhancing its clinical potential. Our supposition is based on biological data which links generation of reactive oxygen species (ROS) to changes in tumor cell metabolism.

Over the last 20 years, multiple investigators have demonstrated that generation of ROS plays a crucial role in radiation cytotoxicity by inducing DNA damage and triggering tumor cell death [13], [14]. Although most studies have focused on physio-chemically generated ROS during IR, our group has recently demonstrated that ROS generated metabolically, in response to IR play an important role in radiation cytotoxicity [15], [16]. Specifically, we have shown that relative levels of metabolically driven ROS correlate with relative radiosensitivity/radioresistance, and that exogenous manipulation of post-IR ROS levels can dramatically alter tumor cell response to IR [15], [16]. Tumor cells maintain substantial levels of glutathione and other thiol-containing proteins, which can scavenge IR, generated ROS [17], [18], [19], [20], [21]. Regeneration of these thiol moieties requires utilization of secondary reducing equivalents such as NAD(P)H, which are generated metabolically [17], [18]. The ability of tumor cells to neutralize IR induced ROS through reducing equivalents is therefore an important modulator of IR cytotoxicity.

Recent advances in hyperpolarized MR tracers such as [1-^13^C]-pyruvate allow measurement of *in vivo* processes that were previously impossible [22], [23]. We and others have shown that it is possible to ascertain in real-time the metabolic activity of solid tumors by measuring conversion of exogenous pyruvate into lactate [24], [25]. Since this conversion requires reducing equivalents in the form of NADH, it can represent an indirect measurement of tumor reducing potential. Indeed, when tumor reducing potential is decreased using metabolic inhibitors (inhibitors of glucose catabolism), conversion rates decrease significantly [25], [26], [27]. Since tumor reducing potential and tumor ROS levels are inversely related, we hypothesized that HP-MRS/MRSI can be used to detect a change in the conversion of pyruvate into lactate following acute exposure to IR.

We chose to test this hypothesis in the context of anaplastic thyroid cancer (ATC), one of the deadliest solid tumors encountered in the head and neck region [1], [2], [4], [28]. IR is commonly employed in the treatment of ATC either as definitive treatment or following surgical resection, in order to increase locoregional control [28]. Death from ATC locoregional disease occurs through festering non-healing wounds, carotid blowouts and suffocation. Because ATC progression is rapid, it is essential to develop more effective IR algorithms for this disease by adjusting treatment in real-time to improve clinical effectiveness.

## Materials and Methods

### Cells

Previously described ATC cell lines were obtained from an established cell bank in the laboratory of Dr. Jeffrey N Myers (University of Texas MD Anderson Cancer Center) under approved institutional protocols. All cell lines were tested and authenticated using short tandem repeat analysis [29]. These cells lines have been previously used by our group and others to evaluate ATC pathogenesis and response to treatment [25], [30]. Wherever possible, we utilized multiple cell lines for *in vitro* experiments to determine whether our findings are relatively generalizable. Cells were maintained in either RPMI of MEM growth media supplemented with glutamine, pyruvate, penicillin/streptomycin and 10% fetal bovine serum.

### Chemicals

2-deoxyglucose, hydrogen peroxide and N-acetyl cysteine (NAC) were purchased from Sigma-Aldrich, (St. Louis, MO). D-glucose was purchased from ICN Biomedical (Irvine, CA).

### Metabolic Studies

Reactive oxygen species (ROS) and cellular reducing potential were measured as previously described [15], [16]. Briefly, total ROS levels were measured using the cell-permeant 2′,7′-dichlorodihydrofluorescein diacetate (H_2_DCFDA). Fluorescence following exposure to specific drugs and or IR was measured using either flow cytometry or spectrophotometrically according to previously published and validated protocols [15], [31]. Cells were pre-treated with ROS modulating drugs for 1 hour prior to irradiation and ROS levels were measured 15′ post IR exposure. Reducing potential was measured using a previously published modified version of the MTT (3-(4,5-dimethylthiazol-2-yl)-2,5-diphenyltetrazolium bromide) to formazan reaction [15], [25], [32]. Briefly, cells were exposed to specific drugs for 0.5–2 hours. Drugs were removed and cells were exposed to the MTT reagent. Following conversion of MTT to formazan, the reaction was terminated and the extent of the conversion was measured spectrophotometrically. For lactate measurements cells (1×10^5^/well) were plated in 12 well plates and irradiated to various doses. Cells were harvested at 0, 15, 30, 60, 120 minutes post-irradiation using lactate assay buffer (BioVision, Milpitas, CA) and snap-frozen in liquid nitrogen. Lactate levels were analyzed by colorimetric assays using a commercially available lactate assay kit (BioVision, Milpitas, CA), according to the manufacturer’s instructions.

### Cytotoxicity Studies

Drug and IR cytotoxicity were assayed using clonogenic assays. ATC cells were irradiated using a high dose-rate ^137^Cs unit (4.5 Gy/min) to the indicated dose. Cells were incubated with drugs of interest for 24 hours; drugs were then removed and cells were incubated for colony formation in fresh media for 10–14 days, then fixed and stained using a 1% formalin/crystal violet solution. Colonies were counted and surviving fractions were determined based upon the plating efficiency of the non-irradiated control group.

### Orthotopic ATC Tumors

Male athymic nude mice (8–12 weeks) were purchased from the National Cancer Institute (Bethesda, MD), maintained in a pathogen-free facility and fed irradiated mouse chow and autoclaved, reverse osmosis treated water. The animal facility was approved by the American Association for the Accreditation of Laboratory Animal Care and met all current regulations and standards of the U.S. Department of Agriculture, U.S. Department of Health and Human Services and the National Institutes of Health. All procedures were approved by the Institutional Animal Care and Use Committee of The University of Texas M.D. Anderson Cancer Center. U-HTH83 luciferase expressing cells (2.5×10^5^/mouse) were injected into the right thyroid lobe under direct visualization as previously described [30].

Tumor size was ascertained regularly throughout the experimental period using bioluminescence imaging as previously described [30]. Tumors were allowed to grow for 1 week prior to initiation of imaging experiments. ATC tumors were irradiated to indicated doses using either a Co^60^ irradiator and custom lead blocks or an image-guided radiotherapy system (X-Rad 225Cx, Precision X-Ray Inc., North Branford, CT). Both irradiation methods delivered the same targeted radiation dose to the same tumor volume. For acute irradiation imaging experiments animals were imaged once to obtain baseline metabolic activity 1.5 hours prior to irradiation and a second time approximately 40 minutes following irradiation with approximately a 2 hour time interval separating the two measurements. Using anatomic T2 weighted images, tumors were measured in 3 dimensions and the volume was defined as *V* = (4/3)π*XYZ*, in which *X*, *Y*, and *Z* represent the radius of the tumor in each dimension. Following completion of all imaging experiments, all animals were sacrificed and tumors were harvested for histologic evaluation. Briefly, tumors were sectioned, stained using haematoxylin and eosin and analyzed in a blinded fashion by a trained head and neck pathologist (MDW).

### Magnetic Resonance Spectroscopy and Spectroscopic Imaging

Hyperpolarized [1-^13^C] pyruvic acid was prepared to a final solution containing 80 mM pyruvate with a nominal pH of 7.6 and temperature of 37°C as previously described [22], [25]. Dynamic spectroscopic data was acquired at 7T using a Biospec USR7030 system and B-GA12 imaging gradients (BrukerBiospin Corp, Billerica, MA) as previously described [25]. Hyperpolarized pyruvate is converted into hyperpolarized lactate through interactions with intracellular coenzymes NADH and lactate dehydrogenase (LDH). Dependence of the pyruvate to lactate conversion on NADH concentration was tested inside of a phantom system prepared from Ultem resin stock with a 3 mL cavity in the center [33]. Polarized pyruvate was injected into the phantom and followed by an enzyme mixture to flush all reagents into the phantom cavity. The combination nominally included 2 mM hyperpolarized ^13^C-Pyruvate, 40 mM Lactate, and 3.92 U/mL Lactate Dehydrogenase (Worthington Biochemical Corp., Lakewood, NJ) in a Tris buffer (81.3 mM Trisma preset crystals pH 7.2, 203.3 mM NaCl) (Sigma-Aldrich, St. Louis, MO). Conversion of HP pyruvate to lactate was measured in 3 separate scans using 0.0, 1.0 and 4.0 mM NADH (Sigma-Aldrich, St. Louis, MO).

For all *in vivo* experiments, anesthesia was induced and maintained using 0.5–5% isoflurane in oxygen. A surface coil (20 mm outer diameter) that was tuned for ^13^C was placed over the thyroid. Animal positioning was confirmed using a 3-plane fast low-angle shot (FLASH) gradient-echo sequence (TE = 3.6 ms, TR = 100 ms) and the location of the tumor was observed in T2-weighted axial and coronal spin-echo images (TE = 50 ms, TR = 2500 ms, with echo train length of 8, 4 cm×3 cm FOV encoded over a 256×192 matrix, and 1 mm slice thickness). For ^13^C measurements, signal was excited using the ^13^C channel of the dual-tuned resonator and detected using the surface coil. Dynamic spectra were acquired every 2 s for 3 min using a slice-selective pulse-acquire sequence (TE = 2.4 ms, TR = 2 s, 10° excitation angle, 2048 points acquired over a 4.96 kHz bandwidth) beginning just prior to injection of 200 µl of 80 mM hyperpolarized pyruvate solution via tail vein catheter. Spectra were phase-adjusted, and the area of the pyruvate and lactate spectral peaks was integrated to yield a time-intensity curve reflecting the arrival of hyperpolarized pyruvate and its conversion into hyperpolarized lactate. These curves were integrated in time to calculate the total relative amounts of hyperpolarized pyruvate and lactate observed over the course of each experiment, and a normalized measure of lactate was formed by dividing total lactate by the sum of lactate and pyruvate. Spectroscopic images of hyperpolarized pyruvate and lactate were acquired using an echo planar spectroscopic imaging (EPSI) sequence with the following acquisition parameters: TE_1_/TR = 3.9/100 ms, ΔTE = 0.46 ms, 64 echoes, FOV = 3.5×1.75 cm, matrix = 32×16, and a 1 cm slice thickness. The images were collected during a 1.6 s window at 15 s after the tracer injection ended. This time corresponded with the highest expected lactate level, based on time courses obtained from the dynamic HP spectra. Images were reconstructed using Matlab (The Mathworks, Natick, MA).

### Modeling of Pyruvate to Lactate Conversion

The HP signal pool is not renewable and is reduced by intrinsic relaxation processes and losses due to signal excitation. Models can be used to account for these factors and characterize tracer exchange [34]. We used a kinetic model that employs two physical compartments and two chemical pools in order to additionally account for tissue perfusion and further separate chemical exchange from the effects of upstream biological barriers. Signal originating from within the intravascular space is differentiated from extravascular signal as consistent with the widely-used extended Tofts model for tracer kinetics [35], and in this work, it was further assumed that equilibrium between intracellular and extracellular extravascular pyruvate was very rapid resulting in a single equivalent extravascular compartment. Although pyruvate can be converted into multiple metabolites *in vivo*, because our interest is in the conversion of pyruvate to lactate and its dependence on intracellular reducing potential, these were the only two chemical species that were explicitly characterized and included in the kinetic model. The transition of pyruvate through other chemical pathways is accounted for through a variable that reflects spin-lattice relaxation and other indistinguishable routes of clearance. Here, the vascular input function was approximated by a gamma distribution of variable shape and amplitude, and the time-intensity curves measured as described above for HP pyruvate and lactate were fit to the model using Matlab. This approach allowed for estimation of *k_PL_* (rate of pyruvate to lactate conversion), the rate of chemical exchange from intracellular HP pyruvate to lactate, among several complex and potentially confounding factors.

### Statistical Analysis

All *in vitro* experiments were carried out at least in triplicate (for each condition) and were repeated tonsure reproducibility. All statistical analysis for *in vitro* experiments was conducted using two-tailed Student’s t-test analysis with a cutoff p-value of 0.05 to demonstrate statistical significance. For the acute irradiation experiment, 3 control and 4 irradiated tumors were imaged. The generation of labeled lactate (nLac) and conversion rate for labeled pyruvate into lactate (kPL) were measured and compared within and across the two groups. P-values were generated using a two-tailed Student’s t-test. For the chronic irradiation experiment the same parameters were measured for 3 control and 3 irradiated tumors and compared across the two groups. P-values were generated using a two-tailed Student’s t-test.

## Results

### Anaplastic Thyroid Carcinoma Response to Ionizing Radiation

**Figure 1:**
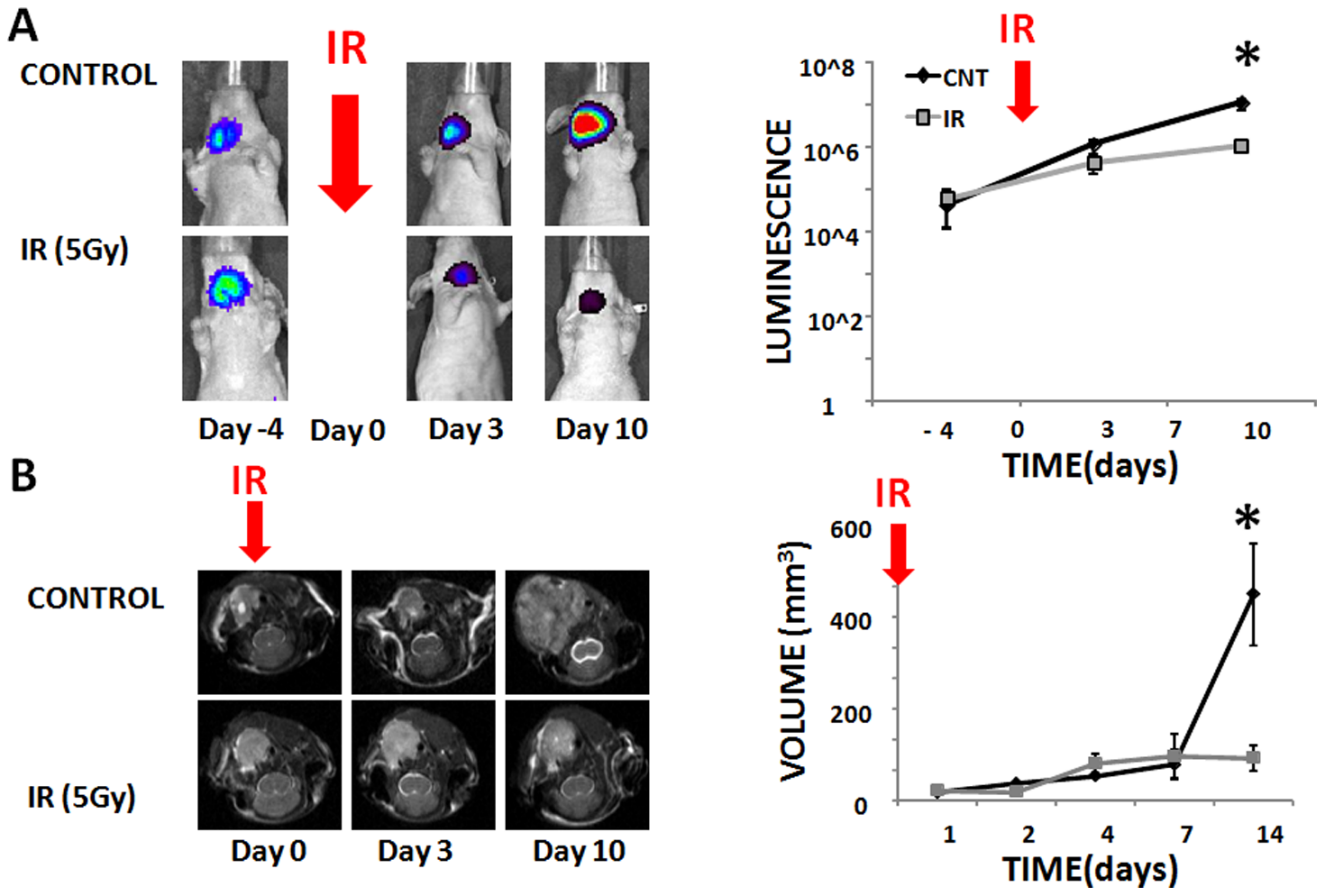
IR decreases but does not arrest ATC growth. A) ATC tumors were allowed to grow under control conditions or following irradiation (IR) (5 Gy single fraction) administered at Day 0. Tumors were imaged using Xenogen imaging prior to and following irradiation (please note y-scale is logarithmic). B) ATC tumors were imaged at multiple points during the experimental period and tumor volume calculated as a product of the largest dimensions in the axial, sagittal and coronal planes. Error bars indicate standard deviation and *denotes p-value <0.05 compared to control for the specific time point using two-tailed Student’s t-test.

To evaluate the effectiveness of ionizing radiation in the single treatment modality setting, orthotopic ATC tumors were generated as described above. Under control conditions, tumor growth was rapid as measured both by luciferase activity (Figure 1A) and anatomic magnetic resonance imaging (MRI) (Figure 1B). Administration of IR in a single fraction of 5 Gy (on Day 0) resulted in a decreased rate of tumor growth but not complete growth arrest (Figure 1). Importantly, as seen in Figure 1B, residual tumor tissue remains essentially unresectable due to involvement of the airway, esophagus and great vessels which is consistent with the clinical progression of the disease in patients. As discussed in a later section, this residual tumor tissue exhibits substantial metabolic activity strongly suggestive of viability. These findings demonstrate the need to better understand ATC tumor response to radiation. As such, we focused our attention on IR induced changes in ROS, a previously demonstrated mechanism of IR cytotoxicity [16], [19].

### Intra-cellular ROS Levels Dictate Response to IR in ATC Cells

**Figure 2:**
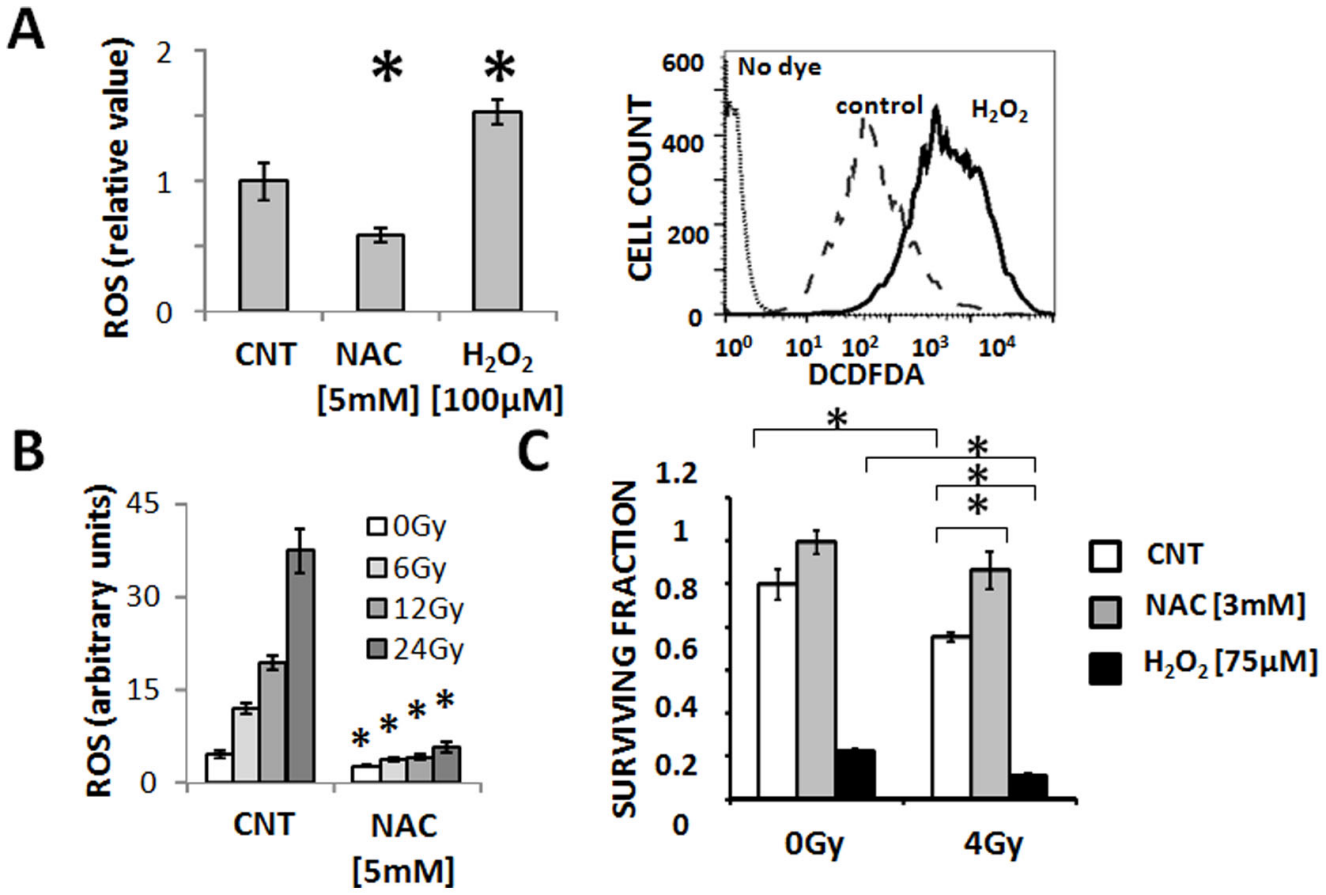
IR cytotoxicity in ATC is driven by changes in ROS levels. A) ATC (U-HTH83) intra-cellular ROS levels can be manipulated through the addition of exogenous ROS sources (H_2_O_2_) or ROS scavenging NAC. B) IR induces a dose dependent increase in intra-cellular ROS levels, which is neutralized by the addition of NAC. C) IR cytoxicity as measured using surviving fraction can be potentiated by the addition of H_2_O_2_ or reversed by NAC. Data are presented as averages with error bars representing standard deviation. Each experiment was performed at least in duplicate. *indicates p-value <0.05 compared to corresponding control condition unless otherwise indicated as in panel C. All experiments were conducted using the U-HTH83 cell line. (CNT = control, DCFDA = 2′,7′-dichlorodihydrofluorescein diacetate).

ATC intra-cellular ROS levels can be exogenously manipulated through the addition of exogenous ROS sources (H_2_O_2_) or scavengers such as N-acetyl cysteine (NAC) (Figure 2A). Exposure of ATC cells to IR induced a dose dependent increase in intra-cellular ROS levels reversed by addition of NAC (Figure 2B). ATC exposure to ionizing radiation decreased ATC cell viability in a dose dependent manner as measured using clonogenic assays (Figure 2C). These effects were exacerbated in the presence of H_2_O_2_ and diminished by the addition of NAC (Figure 2C).

### ATC Cellular Reducing Potential and ROS Levels are Inversely Related

**Figure 3:**
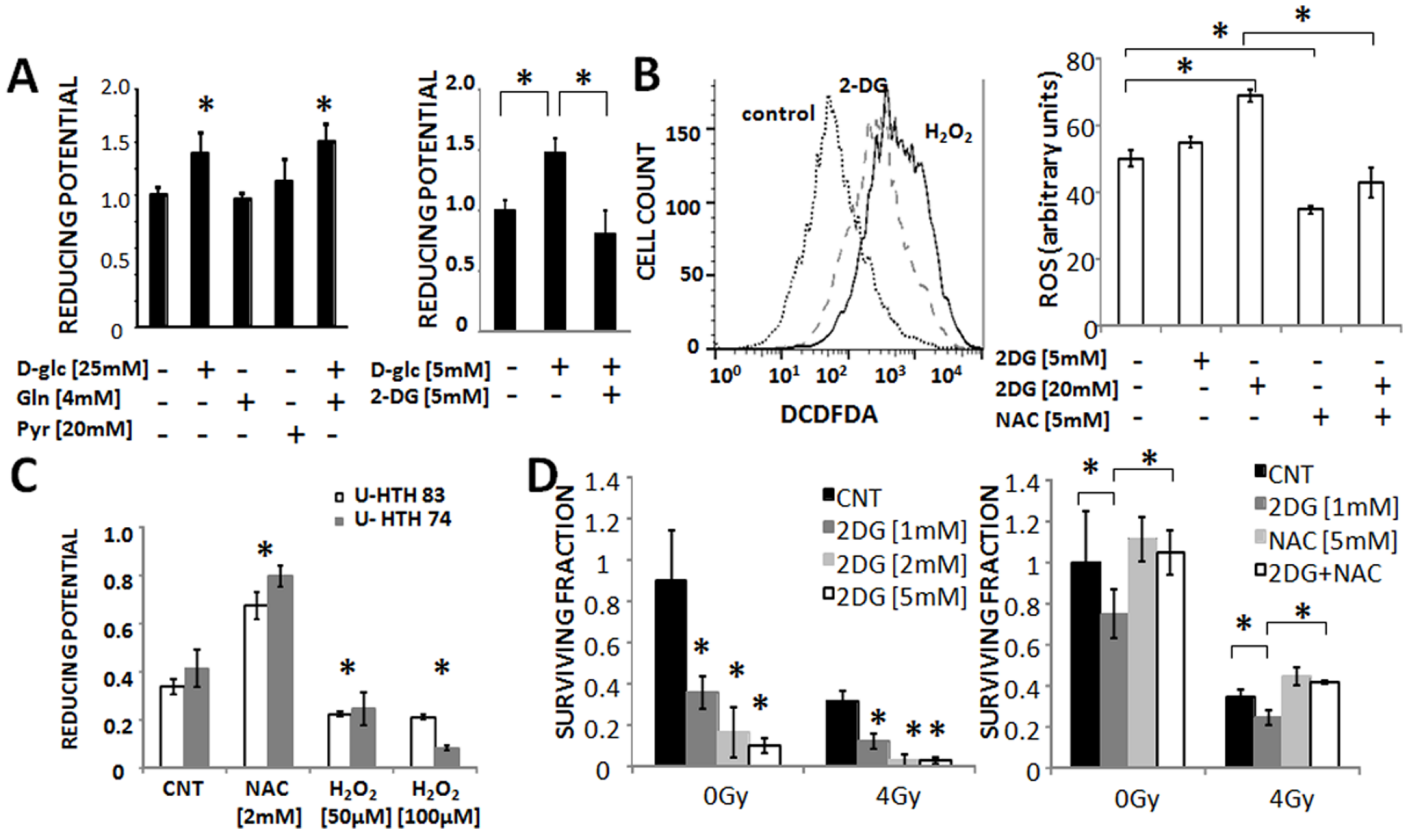
Glucose catabolism controls ATC reducing potential and ROS levels. A) ATC (U-HTH7) cellular reducing potential is maintained largely through glucose catabolism. B) ATC (U-HTH83) inhibition of glucose catabolism using 2-deoxyglucose (2-DG) increases intra-cellular ROS levels in a dose dependent fashion. C) ROS perturbations trigger changes in cellular reducing potential. D) Inhibition of glucose catabolism radiosensitizes ATC cells (U-HTH83) in a dose dependent manner. These effects are reversed by NAC. Data are presented as averages with error bars representing standard deviation. Each experiment was performed at least in duplicate. *indicates p-value <0.05 compared to corresponding control condition unless otherwise indicated. (CNT = control, DCFDA = 2′,7′-dichlorodihydrofluorescein diacetate).

ATC cell reducing potential is primarily driven by the concentration of extra-cellular glucose (Fig. 3A) [25]. Pharmacologic blockade of glucose catabolism using a competitive inhibitor (2-DG) decreased intra-cellular reducing potential (Figure 3A) resulting in a corresponding increase in intra-cellular ROS levels (Figure 3B). Conversely, an increase of intra-cellular ROS levels triggered a corresponding decrease in cellular reducing potential, a phenomenon reversed by NAC (Figure 3C). Decreases in reducing potential using 2-DG resulted in a dose dependent increase in IR cytotoxicity as measured using clonogenic assays (Figure 3D). The effects of 2-DG were reversed by NAC, indicating that 2-DG radio-sensitization is ROS driven. Since ATC reducing potential and intra-cellular ROS levels are inversely related to each other, we hypothesized that measurements of ATC tumor reducing potential can be used to indirectly assay tumor ROS levels.

### HP-MRS can Detect Acute IR Effects on ATC Reducing Potential

**Figure 4:**
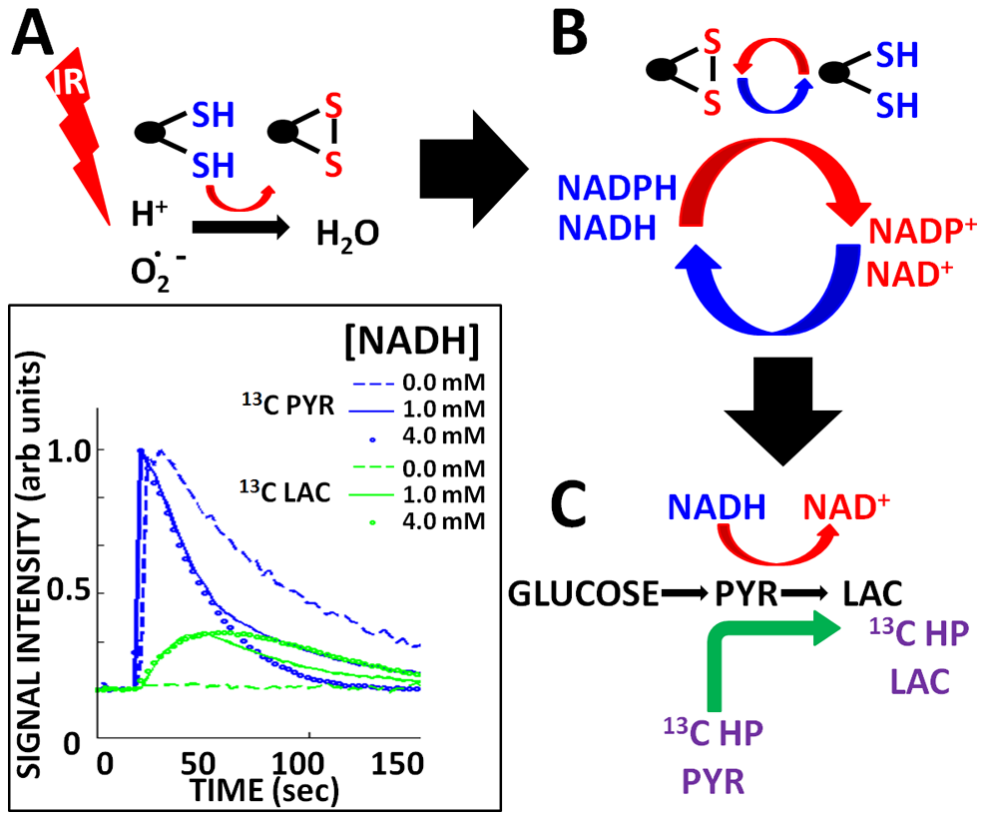
HP-MR detects changes in ROS driven reducing equivalent levels. A) IR increases metabolically driven ROS, which are scavenged by thiol moieties. B) Thiol moieties are regenerated through utilization of secondary reducing equivalents (NADH/NADPH). C) Conversion of endogenous pyruvate into lactate (ubiquitous in tumor cells) requires reducing equivalents. The rate of conversion therefore indirectly reflects global levels of reducing equivalents. HP-MR piggybacks on this endogenous reaction by allowing measurement of conversion of ^13^C pyruvate (PYR) into ^13^C (LAC). The rate of conversion is therefore directly related to reducing equivalent levels and indirectly related to intra-cellular ROS levels. Inset illustrates the dependence of the PYRLAC reaction on available NADH concentration (experiment performed *in vitro* using purified enzyme, NADH and ^13^C HP-PYR).

We have previously demonstrated that it is possible to indirectly measure tumor reducing potential using HP-MRS by measuring the rate of conversion of exogenous ^13^C labeled pyruvate into ^13^C lactate [25]. The dependence of the ^13^C pyruvate to ^13^C lactate reaction on reducing potential is further demonstrated using a synthetic phantom, consisting of substrate (^13^C pyruvate), enzyme and NADH. As shown in Figure 4 (inset) the rate of conversion is dependent on the concentration of NADH.

**Figure 5:**
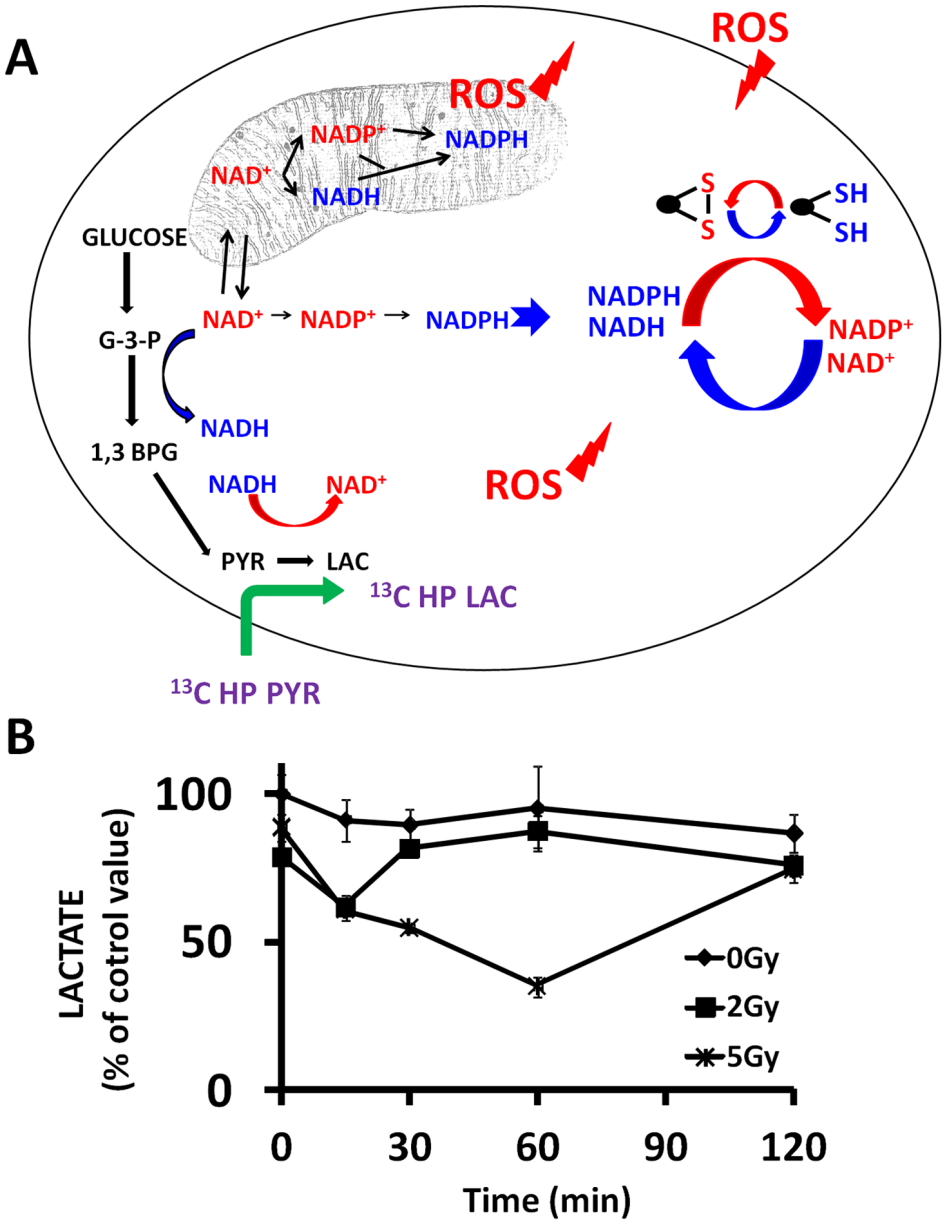
Perturbations in oxidative stress and reducing potential are reflected in altered lactate generation. A) Endogenous reactive oxygen species (ROS) are generated by multiple cellular processes in both the mitochondria and cytoplasm. Exogenous ROS can increase the free radical burden inside the cell. Reducing equivalents in the form of NAD and NADP moieties are generated through multiple metabolic pathways and can cycle rapidly throughout various cellular compartments. Reducing equivalents are utilized by the cell to neutralize ROS. The conversion of pyruvate into lactate requires the presence of NADH; the conversion rate of labeled pyruvate into lactate therefore is an indirect measure of global cellular reducing potential and ROS stress. B) ATC cells were exposed to varying dose of radiation. Cells were harvested at indicated time points and lactate production was assayed biochemically. Data are presented as means, with error bars indicating standard deviation. *indicates p-value compared to control time point <0.05 using a two-tailed Student’s t test.

Since our *in vitro* data demonstrate a reciprocal relationship between tumor cell reducing potential and tumor ROS levels, we hypothesized that exposure to IR will result in a decrease in tumor reducing potential, which could be measured in decreased conversion of exogenous pyruvate into lactate (Figure 4A–C). As shown in Figure 5A, endogenous ROS, generated by mitochondrial activity, or in the cytoplasm, is complemented by exogenous ROS in the form of chemical or radiation induced oxidative stress to generate an overall free radical burden. Tumor cells counteract this oxidative stress through generation of reducing equivalents, which although represented by multiple distinct moieties (NADH, NADPH, etc.) are in fact interchangeable through intra-cellular shuttling and biochemical conversion [36], [37], [38]. As such, we chose to consider cellular reducing potential as a single, global unit. Although somewhat simplistic, considering cellular reducing potential as a global unit is in fact consistent with the application of free radical generating treatment regimens such as ionizing radiation.

The conversion of pyruvate into lactate requires reducing equivalents in the form of NADH. Since IR exposure will deplete reducing equivalents through ROS generation as illustrated in Figure 3, we expected that this would be reflected in decreased cellular lactate production. As shown in Figure 5B, IR exposure triggered rapid and transient drop in lactate production by ATC cells. Interestingly, at lower doses such as 2 Gy the effect is very short lived, and lactate levels return to normal within 15 min following irradiation. At higher doses, the effect is longer lived and the magnitude greater, but lactate levels still return to baseline levels within 2 hours. The time course and transient effect illustrated in this figure essentially exclude events such as cell death, cell cycle arrest, changes in gene expression and protein levels and is completely consistent with our hypothesis, and the relationship between reducing potential and ROS levels illustrated in Figure 3.

**Figure 6:**
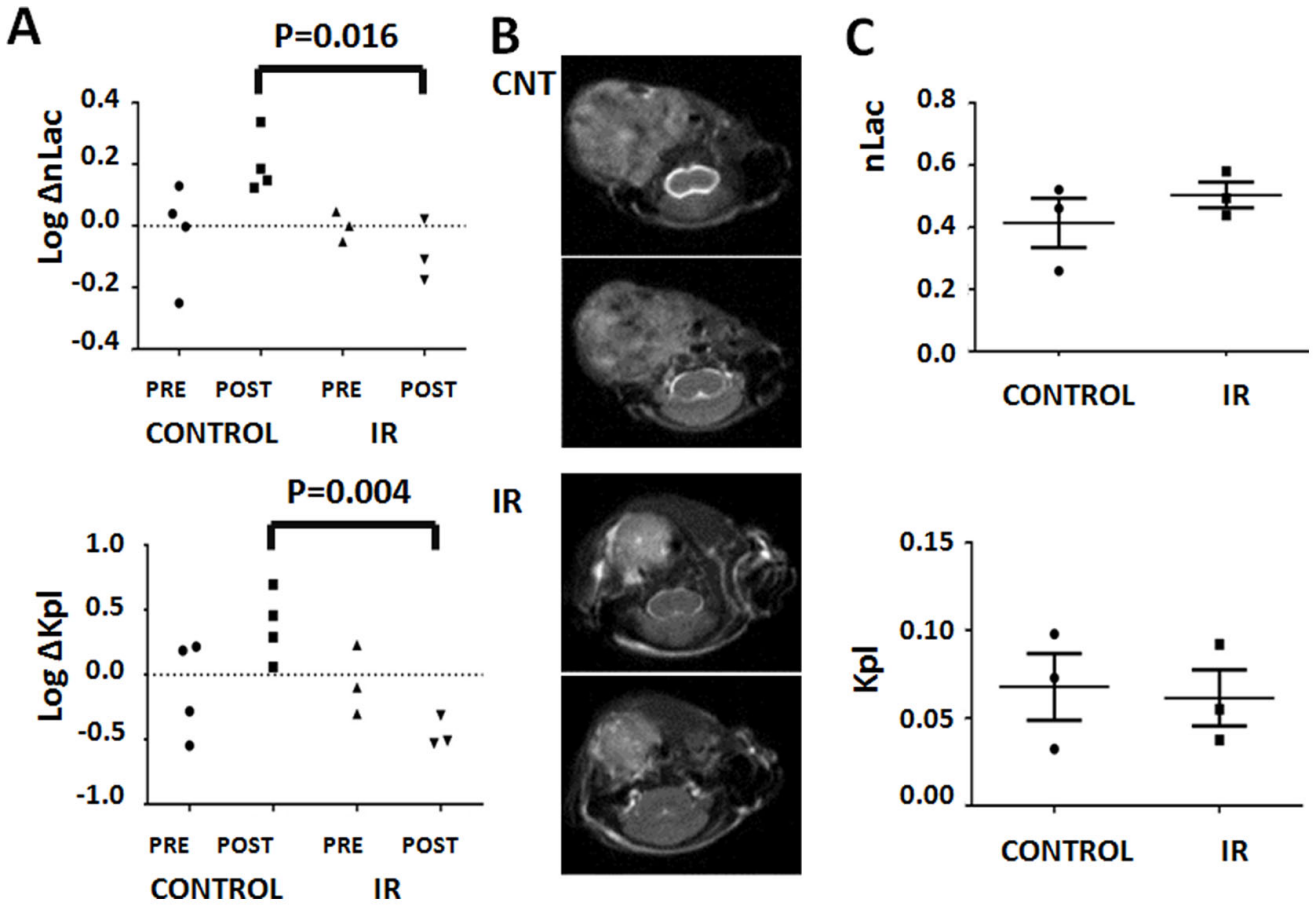
HP-MRS can detect both acute and chronic IR induced changes in ATC reducing potential. A) Control (n = 4) and irradiated (5 Gy) (n = 3) tumors were imaged pre- and post- IR (or sham). Generation of labeled lactate (nLac) and conversion rate constants (Kpl) were calculated and changes from first to second measurement were recorded. B) Control and irradiated ATC tumors demonstrating difference in size using anatomic imaging (T2 weighted sequential slices). C) Reducing potential levels in control (n = 3) and irradiated (n = 3) tumors at 2 weeks post irradiation (5 Gy).

To determine whether these effects can be measured *in vivo* we utilized sequential HP-MRS. Under control conditions, repeated imaging resulted in a consistent increase in both nLac and *k_PL_* between the first and second measurements (Figure 6A). We suspect that this is a function of priming tumors with exogenous pyruvate (which has recently been shown to potentially alter tumor oxygenation) or a side effect of the global physiologic effects of repeated anesthesia [39]. Exposure to a single dose of ionizing radiation resulted in a consistently measurable decrease in conversion of labeled pyruvate into lactate. Compared to the change in control tumors, which were not irradiated between measurements, this change was statistically significant (nLac p = 0.016, *k_PL_* p = 0.004). It is important to note that our experimental design controls for several experimental pitfalls. First, by normalizing post-IR measurements to the pre-IR measurements we account for inter-animal variability. Second, by using repeated imaging in sham irradiated animals, we account for the potential physiologic effects of the imaging tracer described above. Using this dual control, we were able to repeatedly demonstrate a measurable effect of IR on tumor HP-MRS signal. Because repeated measurements were performed within 2 hours of each other, tumor growth, or changes in the rate of growth following irradiation are not likely to be a significant contributor to the changes measured in this experiment.

**Figure 7:**
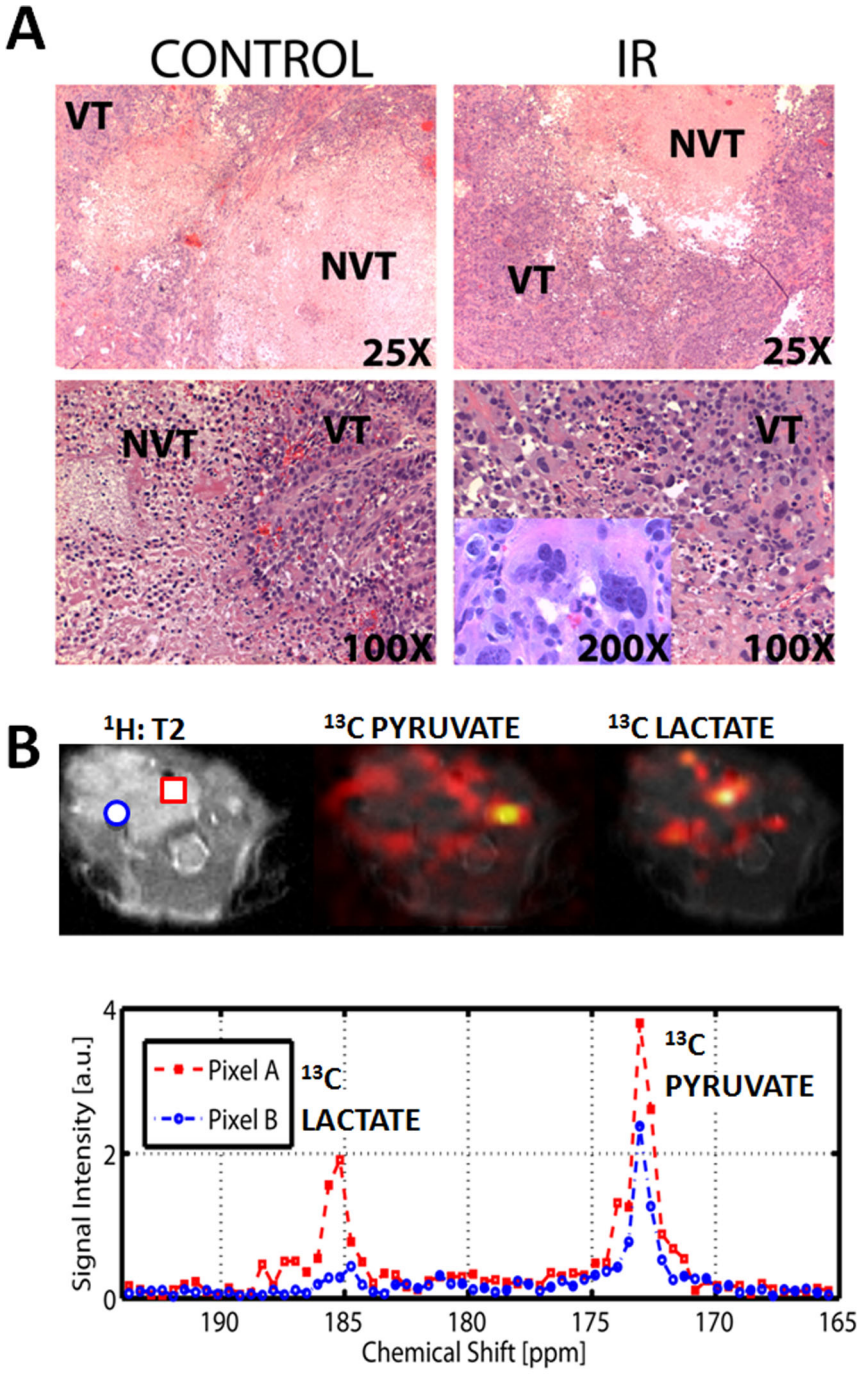
ATC tumors display significant metabolic heterogeneity. A) Control and irradiated tumors (IR) were serially sectioned and H&E staining was used to evaluate overall tumor architecture. Control tumors were substantially larger, but the majority of the tumor core consisted of non viable tissue (NVT). In contrast the majority of the irradiated tumor volume consisted of apparently viable tissue (VT). Irradiated tumors exhibited a high degree of aberrant cellular morphology as illustrated in the right lower panel inset. B) ATC tumor imaged at 2 weeks following tumor cell injection. Single snapshot imaging was performed 20 seconds after injection of labeled pyruvate. Spatial heat maps were generated from raw data and superimposed onto T2 weighted anatomic images for both pyruvate and lactate. Chemical spectra obtained in two separate voxels demonstrating differential conversion of pyruvate into lactate are shown.

Control (n = 3) and irradiated tumors (n = 3) were also imaged at 2 weeks post treatment in order to evaluate chronic effects of IR on ATC tumor reducing potential. Anatomic measurements were made using T2-weighted MRI images (Figure 6B). Control tumor volume was approximately 4 times greater than that of irradiated tumors (Figure 1B, 6B). Despite this significant increase in volume, lactate signal levels for the entire tumor volume were not different between the two groups indicating that the residual volume present in irradiated tumors may be quite viable and metabolically active (Figure 6C). Histologic analysis, illustrated in Figure 7A, indicated that a significant portion of large control tumor volume was occupied by non-viable tissue (mean: 72% necrosis), surrounded by viable tumor cells on the periphery. In contrast the majority of irradiated tumor volume was occupied by apparently viable tissue (mean: 9% necrosis) with a high degree of dysmorphic cellular features. This difference was statistically significant using two-tailed Student’s t-test (p<0.05). The histologic features of irradiated tumors led us to speculate that the larger, un-irradiated tumors might have significant heterogeneity in their reducing potential as measured using HP-MR imaging.

Using previously established imaging sequences, we measured the relative levels of HP pyruvate and lactate in a representative slice through the large control ATC tumor [24]. As shown in Figure 7B, pyruvate signal was maximal in the remaining patent cervical vessels present in the contralateral neck from the xenograft. In contrast, lactate signal was maximal within the tumor itself. However, both lactate and pyruvate signals varied considerably within the tumor volume, with significantly more evidence of pyruvate to lactate conversion on the tumor periphery (Figure 7B). Given the inherent limitations of single snapshot measurements, our findings of metabolic heterogeneity are quite preliminary and will need to be further validated. This additional validation will require the integration of sequences with good temporal and spatial resolution within the HP-MR platform, which is one of the long term aims of our group.

One clinically relevant aspect of the data contained in Figures 6C and 7 is that residual ATC tumor volume at 2 weeks post-irradiation maintains high reducing equivalent levels. Whether this is achieved through cellular adaptation to higher IR-induced ROS levels or selection of radioresistant cells will require additional investigation using both *in vitro* and *in vivo* approaches.

## Discussion

Successful locoregional control of aggressive solid tumors, such as ATC, requires improved ability to monitor radiation effects in real time in order to adjust total dose, dose painting and timing of radiosensitizing agents. Currently, design and implementation of clinical IR protocols are largely empirically driven processes, guided in part by extrapolation of *in vitro* and animal model data. The ability to adjust total dose and dose painting based on real-time, tumor-specific information would represent a paradigm shift in clinical practice and potentially lead to improved clinical effectiveness.

IR cytotoxicity is largely a function of ROS generation [14]. Metabolically driven ROS play an important role in relative radio-sensitivity of head and neck squamous cell carcinoma tumor cells [16]. These findings have been confirmed by other investigators, and are further expanded in this study utilizing a preclinical model of ATC [19], [40]. Since it is currently not technologically feasible to quantitatively measure ROS in solid tumors non-invasively and in real-time, we focused our attention on a surrogate marker, namely tumor reducing potential. We and others have shown that it is possible to ascertain in real-time the relative reducing potential of solid tumors by measuring conversion of exogenous pyruvate into lactate [24], [25]. Since this conversion requires reducing equivalents in the form of NADH, it represents an indirect measurement of tumor reducing potential. Indeed, when tumor reducing potential is decreased using metabolic inhibitors (inhibitors of glucose catabolism), conversion rates decrease significantly which corresponds with known anti-tumorigenic effects for this agent class [19], [20], [25], [27], [41].

We and others have demonstrated an inverse relationship between tumor reducing potential and ROS levels [16], [19]. In this study we demonstrate the clear relationship between these two parameters under *in vitro* conditions. Based on these data, we hypothesized that IR induced ROS increases would result in reciprocal decreases in tumor reducing potential which could be monitored in real time using HP- MRS measurements of pyruvate to lactate conversion. We first demonstrated a rapid, transient, dose dependent decrease in lactate production measured biochemically under *in vitro* conditions. The transient nature of this change is completely consistent with the transient reciprocal perturbations we had previously measured in ROS and reducing potential in ATC cells, and served as additional confirmation that HP-MRS using labeled pyruvate should be able to detect perturbations in tumor reducing potential immediately following irradiation.

Data from two recent studies indicate that HP-MRS can be used to ascertain IR effects on tumor cell death and surrounding tissue toxicity [16], [42]. Our results demonstrate a significant decrease in ATC tumor reducing potential immediately following irradiation, confirming our hypothesis. This novel finding highlights the potential of this imaging modality to monitor IR effects in real-time. Successful demonstration of dose dependent changes and modulation using targeted inhibitors of tumor reducing potential should provide additional information required to develop this into a clinically useful imaging algorithm.

HP-MRS of irradiated tumors demonstrated a second important phenomenon. Although IR exposed ATC tumors exhibited significantly slower growth rates, growth was not completely arrested. Despite smaller tumor volume, irradiated ATC tumors exhibited high reducing potential. This may be a result of two interacting biological phenomena: 1) selection of tumor cells which exhibit increased reducing potential and 2) increasing metabolic heterogeneity associated with large solid tumor volumes (in un-irradiated, control tumors). It is possible that residual tumor tissue following irradiation exhibits a metabolic phenotype required to survive the initial cytotoxic event. This would be consistent with data presented by Albers et al., which demonstrated that increased conversion corresponded to more aggressive solid tumor histology [24]. A second explanation would be that the normal tissue surrounding irradiated tumors contributes a larger percentage of the measured signal. This would be consistent with data which suggests that radiation-induced injury to normal tissue and resulting inflammation can itself lead to significant increases in conversion [42]. Signal localization by tomographic imaging of HP tracer evolution will minimize this ambiguity in future studies.

Improved IR effectiveness in controlling the growth of ATC and other aggressive solid tumors is an important clinical goal. Although investigators have been able to characterize IR effects *in vitro* with exquisite detail, *in vivo* studies have been limited by an inability to monitor acute IR effects on tumors in real-time. For the first time, we provide evidence that such measurements are in fact possible, by monitoring changes in tumor reducing potential using HP-MRS/MRSI. This is the first study to demonstrate acute perturbations in tumor reducing potential following irradiation using a non-invasive means suitable for clinical translation [43]. In addition, it is the first study to demonstrate increased metabolic activity in residual tumor tissue post-IR. Enhanced spatial and temporal resolution through improved dynamic spectroscopic imaging sequences combined with conformal intensity modulated radiation therapy (IMRT) may allow for real-time adjustments in dosimetry maps in order to maximize tumor cytotoxicity. Clearly, significant challenges remain in developing HP-MRS/MRSI into a tool that can *predictably* monitor IR effects in real time. Overcoming these challenges will require the continued development of acquisition, reconstruction, and signal analysis strategies in order to more precisely characterize tumor metabolic changes.
